# Community Structure and Functional Prediction of Gut Microbiota in Mandarin Fish (Siniperca Chuatsi) Under Different Culture and Feeding Conditions

**DOI:** 10.3390/life16071194

**Published:** 2026-07-19

**Authors:** Yayu Wang, Chen Niu, Xiaoyu Jiang, Jinpeng Zhang, Shu Wang, Cuiping Gong, Dayan Hu

**Affiliations:** 1 Huzhou Academy of Agricultural Sciences, Huzhou Municipal Bureau of Agriculture and Rural Affairs, Huzhou 313000, China; wangyayu521@126.com (Y.W.); jxy_lected@163.com (X.J.); kim1103@foxmail.com (J.Z.); wangshu0301@163.com (S.W.); gongcuiping1209@126.com (C.G.); 2 Zhejiang Institute of Freshwater Fisheries, Huzhou 313000, China; 19969595285@163.com

**Keywords:** intestinal microbiota, *Siniperca chuatsi*, artificial feed, breeding environment

## Abstract

This study investigated the effects of culture systems and feed types on the gut microbiota of mandarin fish (*Siniperca chuatsi*), using three production culture modes: pond–live bait (CE), pond–artificial feed (CS), and RAS–artificial feed (GS). Under identical pond culture conditions, CS fish fed compound feed had higher relative abundances of carbohydrate-metabolizing Bacteroidota and Firmicutes than CE. The CS group showed higher α-diversity, but β-diversity analysis indicated no significant community difference (*p* = 0.36), demonstrating that feed replacement only induced mild microbial shifts. LEfSe analysis uncovered 17 differential taxa: homeostasis-related genera enriched in CS, while the lipid-adapted Macellibacteroides prevailed in CE. Under identical artificial feed conditions, switching culture mode from pond (CS) to industrial RAS (GS) drastically reduced gut microbial diversity and simplified community structure. GS exhibited a sharp rise in Fusobacteriota, with Cetobacterium (64.94%) becoming the single dominant genus. α-diversity was lower in GS than CS, and β-diversity confirmed highly distinct community profiles between the two groups (*p* = 0.004). LEfSe detected 43 differential taxa with diverse metabolic genera abundant in CS. No PICRUSt2-inferred KEGG pathways remained significant after FDR correction in the above two comparison groups, so these functional trends are only for reference.

## 1. Introduction

Mandarin fish (*Siniperca chuatsi*) is an economically important freshwater fish species in China, favored by consumers for its tender flesh, delicious taste, and high nutritional value [1]. With the continuous improvement in living standards and the expansion of high-end aquatic product markets, the demand for Mandarin fish has been steadily increasing. According to statistics, as of 2023, China’s production of Mandarin fish has reached 477,600 tons, an increase of 18.95% compared to 2022 [2]. However, the traditional model of Mandarin fish farming is increasingly constrained by multiple challenges. Conventional culture mainly relies on outdoor pond systems and live bait fish feeding, which is associated with high land occupation, low unit yield, unstable bait supply, and increased risk of pathogen transmission from live prey [3]. In addition, pond environments are easily affected by seasons, weather, and external water sources, resulting in unstable water quality and difficulties in standardized management. These problems have collectively restricted the green, healthy, and sustainable development of the mandarin fish industry.

To alleviate these constraints, the mandarin fish aquaculture industry is actively transforming toward intensive and environmentally friendly modes, among which industrial recirculating aquaculture systems (RAS) and artificial compound feed have become two core directions of technological upgrading. RAS is characterized by high-density culture, closed-loop water circulation, stable environmental parameters, and reduced water and land consumption, which can effectively improve production stability and product safety [4]. Meanwhile, artificial compound feed can eliminate dependence on live bait fish, standardize nutrition supply, reduce disease transmission risks, and facilitate large-scale industrial management [5]. The feasibility and advantages of RAS and artificial feed have been verified in many cultured fish species, such as common carp (*Cyprinus carpio*) [6], shrimp (*Penaeus vannamei*) [7], and rainbow trout (Oncorhynchus mykiss) [8], *Micropterus salmoides* [9] as well as *Salmo salar* [10]. However, their comprehensive effects on the physiological status and intestinal health of mandarin fish remain to be systematically elucidated, especially the associations of combined environment and diet with gut microbial homeostasis.

The gut microbiota is closely involved in key physiological processes of fish, including nutrient digestion and absorption, energy metabolism, immune response, and disease resistance [11,12]. Changes in host selection, diet composition, and environmental factors can collectively shape the community structure and functional potential of the gut microbiota [13]. During the transition from traditional pond farming to industrial RAS farming, and from live bait to artificial feed, the farming environment and nutrient intake patterns of mandarin fish have undergone profound changes, which may lead to significant changes in their gut microbiota. Although there have been studies exploring the characteristics of gut microbiota in mandarin fish, there are few studies that simultaneously examine the gut microbiota characteristics under the three mainstream practical production modes covered in this experiment (pond live bait, pond compound feed, and circulating water compound feed). Analyzing the changes in gut microbiota among the representative aquaculture models mentioned above can help reveal the adaptive mechanism of gut microbiota in mandarin fish to industrial aquaculture transformation.

High-throughput sequencing of the 16S rRNA gene provides a robust approach to characterizing microbial responses to environmental and dietary perturbations. Here, we compared gut microbiota across three representative culture groups: pond with live bait (CE), pond with artificial feed (CS), and RAS with artificial feed (GS). The aim of this study is to explore how aquaculture environment and feed type affect the structure, diversity, and predictive function of gut microbiota, and to analyze the correlation between environment and dietary choices and gut microbiota. These findings will deepen our understanding of gut microbiota adaptation in industrial aquaculture and provide scientific references for optimizing feed formulations and promoting the healthy aquaculture of mandarin fish.

## 2. Materials and Methods

### 2.1. Experimental Samples Collection

The experimental site is located in Huzhou City, China (28.3752° N, 116.9917° E). The experimental samples were collected from Wu’s Ecological Agriculture Co., Ltd (Huzhou, China). All fish were acclimated under identical conditions prior to the trial, then randomly allocated to three main production modes: CE, CS and GS. The initial fish size and health status were standardized across all groups. The initial weight of mandarin fish is 2.62 ± 0.67 g (Mean ± SD). They were domesticated at a density of 2000 fish/m^3^; the average size after domestication was 16.12 ± 0.81 g. They were then cultured in three experimental groups. Each mode contained three independent replicated units. The CE and CS groups each use three independent standardized outdoor soil ponds, with a size of 60 × 20 m and a water depth of 1.5 m. During the breeding period, dissolved oxygen is greater than 5.0 mg/L, pH 7–8, ammonia nitrogen is less than 1.2 mg/L, nitrite is less than 0.15 mg/L, and the stocking density is 3 fish/m^3^. The GS group uses three completely independent circulating water aquaculture systems, and the RAS water tank is a cylindrical water tank with a capacity of 7 m^3^. During the breeding period, dissolved oxygen is greater than 6.0 mg/L, pH 7–8, ammonia nitrogen is less than 1.2 mg/L, nitrite is less than 2.0 mg/L, and the stocking density is 100 fish/m^3^. The crude protein, crude fat, crude fiber, ash content, total phosphorus, lysine, and moisture content of artificial compound feed were 47%, 6%, 5%, 21%, 1%, 3.1%, and 12%, respectively. After a 12-month feeding period, 2 healthy mandarin fish were randomly collected from each independent breeding unit, and a total of 6 biological replicates were obtained from each group (n = 6 per group; n = 18 in total). The final body weights of the CE group, CS group, and GS group were 481.06 ± 45.01 g, 445.15 ± 26.66 g, and 656 ± 34.24 g, respectively.

All fish were fasted for 24 h before sampling, and anesthetized with eugenol. The intestinal contents of each fish were collected and sequenced separately as independent biological samples. The entire intestinal tract was dissected under sterile conditions, and the gut contents were collected into sterile cryovials, and then immediately frozen in liquid nitrogen, and stored at −80 °C until DNA extraction. No significant differences in mortality or health status were observed among groups during the trial.

### 2.2. Feed Composition and Feeding Management

Two feeding regimens were used in this study: live bait fish and artificial compound feed. Live bait fish were freshly obtained from the same source throughout the experiment, and fed to the CE group twice daily to apparent satiation. The artificial compound feed used in CS and GS groups was a commercial diet formulated specifically for mandarin fish (Jie Da Company’s Puffed Mandarin Fish Compound Feed). Fish in the CS and GS groups were fed the artificial diet twice daily, adjusted based on observed consumption. The feeding management within each group ensures consistency.

### 2.3. DNA Extraction, Library Construction and Sequencing

Total genomic DNA was extracted using MagPure Soil DNA LQ Kit (Magan, Guangzhou, China, D6356-02) following the manufacturer’s instructions. DNA concentration and integrity were measured with NanoDrop 2000 (Thermo Fisher Scientific, Wilmington, DE, USA) and agarose gel electrophoresis. Extracted DNA was stored at −20 °C until further processing. The extracted DNA was used as a template for the PCR amplification of bacterial *16S rRNA* genes with the barcoded primers and Takara Ex Taq (Takara, Dalian, China). For bacterial diversity analysis, V3-V4 variable regions of 16S rRNA genes was amplified with universal primers 343F (5′-TACGGRAGGCAGCAG-3′) and 798R (5′-AGGGTATCTAATCCT-3′) [14]. The Amplicon quality was visualized using agarose gel electrophoresis. The PCR products were purified with AMPure XP beads (Beckman Coulter, Beverly, MA, USA) and amplified for another round of PCR. After purification with the AMPure XP beads again, the final amplicon was quantified using Qubit dsDNA Assay Kit (Thermo Fisher Scientific, Eugene, OR, USA). The concentrations were then adjusted for sequencing. Sequencing was performed on an Illumina NovaSeq 6000 with 250 bp paired-end reads. (Illumina Inc., San Diego, CA, USA; OE Biotech Company; Shanghai, China). The library sequencing and data processing were conducted by OE biotech Co., Ltd. (Shanghai, China). The raw data was submitted to the NCBI database, with the submission accession number PRJNA1423420.

### 2.4. Analysis of Sequencing Data

Paired-end reads were preprocessed using Cutadapt software v4.4 to detect and cut off the adapter. After trimming, paired-end reads were filtering low-quality sequences, denoised, and merged, and chimera reads were detected and cut off using DADA2 [15] of QIIME2 (2020.11) [16]. The specific parameters are as follows: --p-trim-left-f: 0, --p-trim-left-r: 0, --p-trunc-len-f: 0, --p-trunc-len-r: 0, --p-max-ee-f: 2, --p-max-ee-r: 2, --p-trunc-q: 2, --p-min-overlap: 12. For chimera filtering, the parameter --p-min-fold-parent-over-abundance was set to 1.0, and --p-allow-one-off was False. After denoising, ASV table and representative sequences were obtained. It should be noted that no blank negative-control samples were set during DNA extraction and library construction in this study. These were normalized by taking the minimum number of reads from all samples, dividing the number of species reads by the total, and standardizing to a total of 1. Taxonomic classification of representative ASVs was carried out against the SILVA 138.1 database. QIIME 2 software (University of California, San Diego, CA, USA) was used for α and β diversity analyses. The microbial diversity in samples was estimated using the alpha diversity, which included ACE, Chao1 [17], Shannon [18] and Simpson [19]. The Euclidean distance matrix performed by R package was used for Principal coordinates analysis (PCoA) to estimate the similarities or differences between bacterial communities. Adonis analysis was performed using the compact_categorys.tpy tool in QIIME, and the Adonis difference *p*-value was calculated using the Euclidean distance algorithm. A *p*-value of *p* < 0.05 is considered significant. LEfSe analysis reveals the composition of species differences between two or more biological communities.

### 2.5. Predictive Functional Analysis of Bacterial Communities

PICRUSt2 v2.4.2 (Phylogenetic Investigation of Communities by Reconstruction of Unobserved States) [20,21] software predicts the composition of known microbial gene functions in order to statistically analyze the functional differences between different groups. Functional prediction was performed based on the 16S KEGG database and a statistical analysis was performed on the predicted KEGG results at three levels using the t-test algorithm. Microbial differences between the two groups were determined based on significance (*p* < 0.05) and a heatmap of the different species was drawn. The mean NSTI value of all samples was 0.3507 ± 0.31, which exceeded the recommended threshold of 0.25 and indicated the relatively low reliability of PICRUSt2 functional inference. Therefore, all KEGG pathway results were only treated as auxiliary reference rather than evidence of actual microbial metabolic activities.

### 2.6. Statistical Analysis

All values were reported as means ± standard errors (SEM), and SPSS 22 was used for data analysis. An independent samples *t*-test was employed to identify the differences in growth performance between the two groups. Significance was determined at a threshold of *p* < 0.05.

## 3. Results

### 3.1. 16S rRNA Sequencing Results

**Figure 1:**
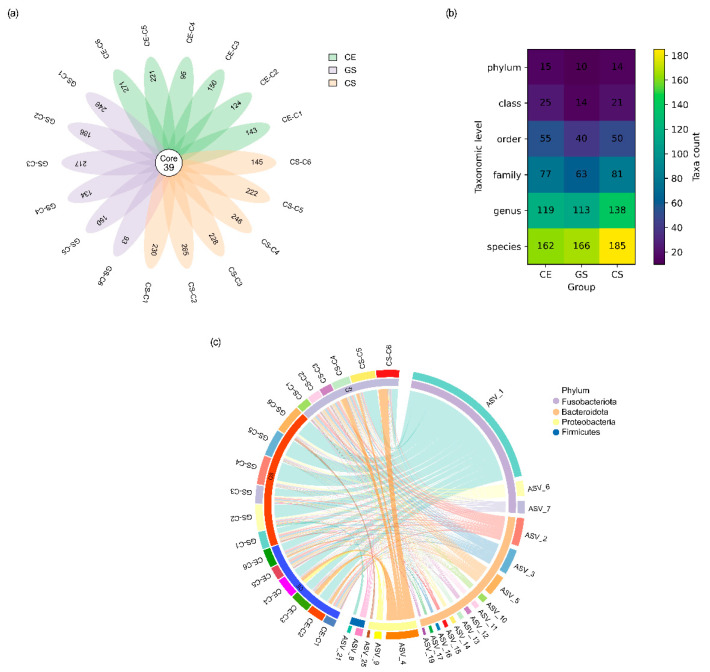
(**a**) The Venn diagram of ASV distribution in three groups, with the number of ASVs in each sample labeled in the figure. (**b**) Statistical analysis of gut microbiota at different levels within each group. (**c**) Circos plot illustrating the correlation between the three groups and the four dominant bacterial phyla, as well as their corresponding characteristic ASVs.

This study performed a double-ended amplicon 16S rRNA gene sequencing based on the NovaSeq Illumina platform. The raw reads data volume of the sequencing machine ranges from 78,031 to 81,744, the clean tags data volume after quality control ranges from 49,176 to 72,421, the valid tags data volume obtained by removing chimeras from clean tags ranges from 36,883 to 65,224, and the number of ASVs in each sample ranges from 132 to 310. In order to further analyze the distribution characteristics of ASVs among different samples, we created a flower chart that visually displays the number of ASVs within each sample and the ASVs shared among the three groups. A total of 39 core ASVs were shared across the three groups (Figure 1a). Taxonomic annotation identified 16 phyla, 32 classes, 72 orders, 112 families, 187 genera, and 276 species in the gut microbiota. The identification of each group is shown in Figure 1b. Circos described the correspondence between three aquaculture groups and four dominant bacterial phyla, especially the relationship with the characteristic expression sequence ASV (Figure 1c).

### 3.2. Gut Bacterial Composition

**Figure 2:**
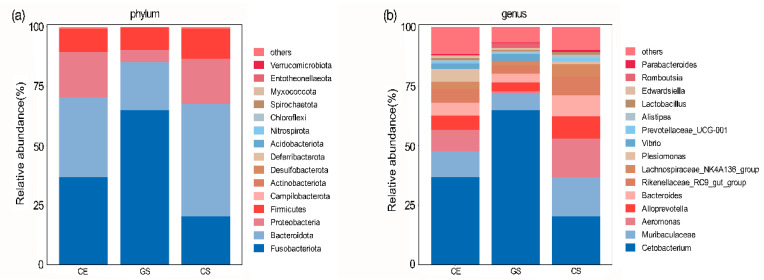
(**a**) Bar chart of community structure for three groups (phylum). (**b**) Bar chart of community structure for three groups (genus).

A bar chart illustrating the top 15 most abundant species at both the phylum and genus levels was generated for the three groups. At the phylum level, Fusobacteriota, Bacteroidetes, Proteobacteria, and Firmicutes consistently exceeded 5% relative abundance across all groups, though their distributions varied. Their respective abundances were 36.98%, 33.43%, 19.10%, and 9.96% in the CE group; 65.00%, 20.25%, 5.14%, and 9.25% in the GS group; and 20.49%, 46.99%, 19.07%, and 12.90% in the CS group (Figure 2a). At the genus level, the number of taxa with relative abundance exceeding 5% varies among the three groups. The CE group contained the highest number (seven genera), predominantly Cetobacterium (36.86%), Muribaculaceae (10.84%). In the SILVA 138.1 database, when there is no known formal genus name at the genus level, the database automatically fills in the family name to the genus hierarchy. In this article, Muribaculaceae represents an unnamed genus in the Muribaculaceae family), Aeromonas (8.90%), Alloprevotella (6.03%), Bacteroides (5.53%), Rikenellaceae_RC9_gut_group (5.61%) and Plesiomonas (5.35%). The CS group ranked second, with six genera exceeding the 5% threshold: Cetobacterium (20.34%), Muribaculaceae (16.33%), Aeromonas (16.41%), Alloprevotella (9.36%), Bacteroides (8.77%), and Rikenellaceae_RC9_gut_group (8.1%). The GS group had the fewest, with only two genera above this level: Cetobacterium (64.94%) and Muribaculaceae (7.27%) (Figure 2b). The relative abundance of dominant bacterial genera shared by the three groups has undergone significant changes, with the microbial community of the GS group presenting a relatively simple structure dominated by Cetobacterium, while the community composition of CE and CS groups was more balanced.

### 3.3. Analysis of Alpha and Beta Diversity of Gut Microbiota

**Figure 3:**
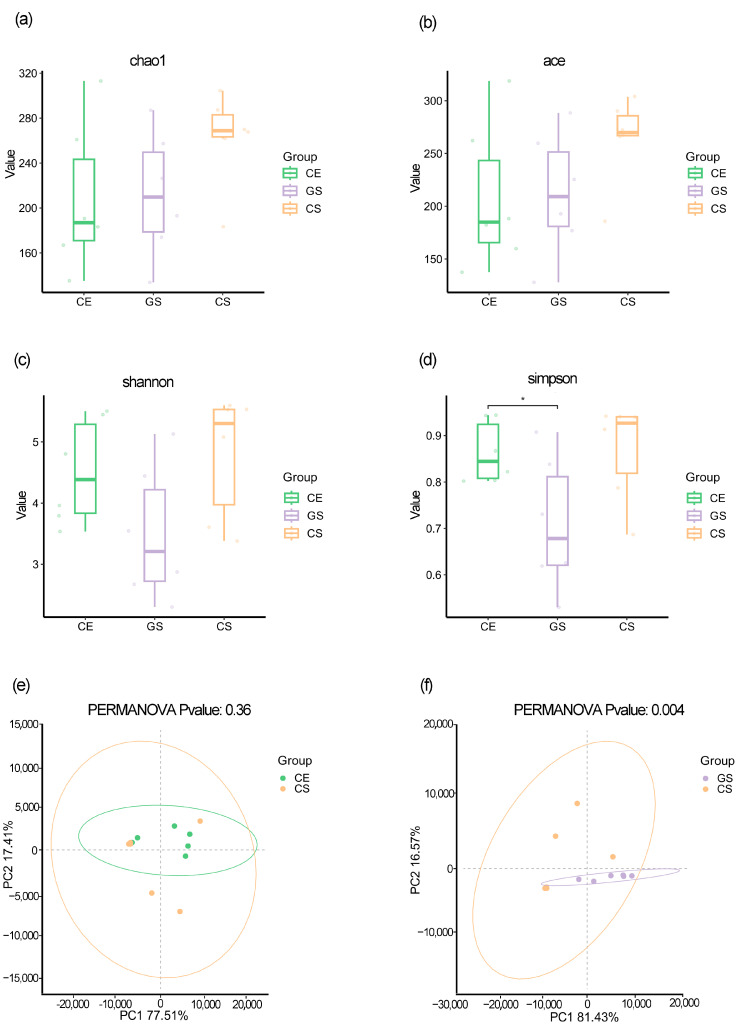
The diversity index of intestinal bacteria in three groups: (**a**) Chao1 analysis; (**b**) Ace analysis; (**c**) Simpson analysis; (**d**) Shannon analysis; (**e**) PCoA analysis; (**f**) PERMANOVA analysis. The asterisk above the bar chart indicates significant differences between groups (*p* < 0.05).

Chao1, Ace, Shannon, and Simpson indices were used to analyze the alpha diversity of gut microbiota, which were used to evaluate species richness and diversity, respectively (Table 1). The CS group had the highest values of all four indices, indicating higher microbial richness and community diversity. The Shannon index and Simpson index of the CE group were similar to those of the CS group, indicating high diversity. In contrast, the Shannon index and Simpson index of the GS group were the lowest, indicating that their community diversity and evenness were the most limited among the three groups. The T.TEST method was used to compare and analyze the differences between each two groups, and the results showed that there was a significant difference in Simpson index between the CE and GS groups (*p* = 0.048), while there was no significant difference between other indices and groups (*p* > 0.05) (Figure 3a–d).

In terms of β diversity, PCoA and PERMANOVA (Adonis) analysis based on Euclidean distance showed that there was no dispersion trend in the PCoA plot between the CE vs. CS groups. The Adonis test further confirmed that there was no significant difference in microbial community structure between the two groups (*p* = 0.36) (Figure 3e). In the GS vs. CS group, there is a trend of concentrated aggregation of GS and the *p*-value is 0.004 (Figure 3f). There is a significant difference between the two groups.

### 3.4. Analysis of Gut Microbiota Differences Related to Environment and Feed

**Figure 4:**
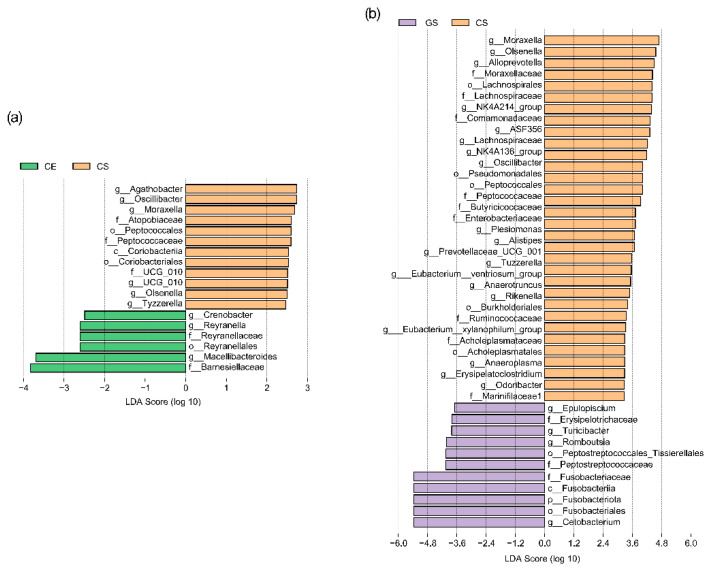
LEfSe analysis: (**a**) CE vs. CS groups; (**b**) GS and CS groups. The colors of the bar chart represent the respective groups, and the length represents the LDA score, which represents the degree of significant differences in the impact of species between different groups.

LEfSe analysis was used to compare differences between groups, including an analysis of the contribution of different species to differences, an annotation analysis of different species, and calculation of the relative abundance of different species in each sample. There are 17 differences between the CE group and the CS group, including 3 orders (starting with o_), 5 families (starting with f_), and 9 genera (starting with g_). The genus-level results showed that Agathobacter, Oscillibacter, Moraxella, UCG_010, Olsenella, and Tyzzerla were highly expressed in CS, while Macellibacteroides, Reyranella and Crenobacter were highly expressed in the CE group (Figure 4a). Comparing the GS and CS groups, there is a significant difference in the number of microorganisms between the two groups, with a total of 43, including 1 phylum (starting with p_, Fusobacteriota), 1 class (starting with c_, Fusobacteriia), 7 orders, 12 families, and 22 genera. Among them, 18 genera are highly expressed in the CS group and 4 genera are highly expressed in the GS group. The top 10 microorganisms with differences in genus level are: Cetobacterium, Moraxella, Olsenella, Alloprevotella, NK4A214_group, ASF356, Lachnospiraceae_NK4A136_group, Oscillibacter, Romboutsia and Turicibacter (Figure 4b).

### 3.5. Changes in Intestinal Microbial Function

**Figure 5:**
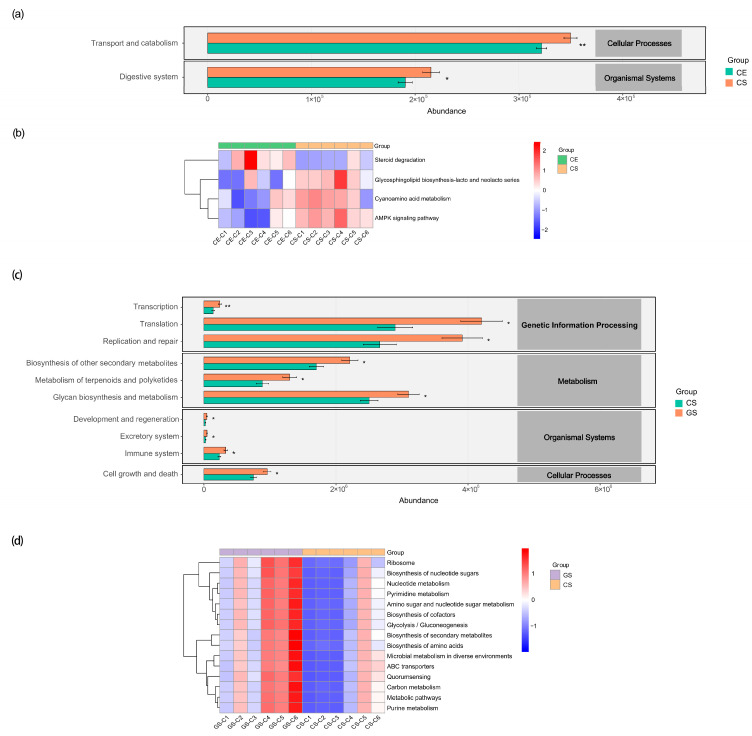
(**a**) Bar chart showing the difference in KEGG functional prediction Level 2 between the CE and CS groups. (**b**) Cluster top-15 heatmap of enriched pathways at Level 3 predicted by KEGG function for CE and CS groups. (**c**) Bar chart showing the difference in KEGG functional prediction Level 2 between the GS and CS groups. * represents *p* < 0.05; ** represents *p* < 0.01. (**d**) Cluster top-15 heatmap of enriched pathways at Level 3 predicted by KEGG function for GS and CS groups.

Picrust2 predictions for the two comparison groups. For the comparison between CE and CS groups, Level 2 KEGG pathways were related to substance transport and catabolism (*p* = 6.23 × 10^−3^, FDR_*p* = 0.280, effect size = −1.885) and digestive system (*p* = 4.12 × 10^−2^, FDR_*p* = 0.859, effect size = −1.242) functions were relatively enriched in the CS group (Figure 5a). Four significantly enriched Level 3 pathways were identified, including the AMPK signaling pathway (*p* = 3.11 × 10^−3^; FDR_*p* = 0.955; effect size = −2.298), glycosphingolipid biosynthesis-lacto and neolacto series (*p* = 1.61 × 10^−2^; FDR_*p* = 0.955; effect size = −1.726), steroid degradation (*p* = 2.96 × 10^−2^; FDR_*p* = 0.955; effect size = 1.847), and cyanoamino acid metabolism (*p* = 4.77 × 10^−2^; FDR_*p* = 0.955; effect size = −1.176) (Figure 5b). Such variations in microbial functions were correlated with different feeding regimens.

For GS and CS, significant differences were observed in Level 1 KEGG pathways associated with metabolism (*p* = 3.16 × 10^−2^; FDR_*p* = 0.095; effect size= −1.489) and genetic information processing (*p* = 1.27 × 10^−2^; FDR_*p* = 0.076; effect size = −1.796) at Level 1. The top five differential Level 2 pathways included transcription (*p* = 9.32 × 10^−3^; FDR_*p* = 0.075; effect size= −1.880), translation (*p* = 1.02 × 10^−2^; FDR_*p* = 0.075; effect size= −1.865), replication and repair (*p* = 1.08 × 10^−2^; FDR_*p* = 0.075; effect size= −1.856), the biosynthesis of other secondary metabolites (*p* = 1.17 × 10^−2^; FDR_*p* = 0.075; effect size = −1.826) and development and regeneration (*p* = 1.42 × 10^−2^; FDR_*p* = 0.075; effect size= −1.732) (Figure 5c). Looking further at the results of the Level 3 pathway, the heatmap shows the top 15 significant differences, among which the top 6 significant differences are the biosynthesis of cofactors (*p* = 1.9 × 10^−2^; FDR_*p* = 0.075; effect size= −1.648), biosynthesis of amino acids (*p* = 2.9 × 10^−2^; FDR_*p* = 0.084; effect size= −1.548), metabolic pathways (*p* = 3.1 × 10^−2^; FDR_*p* = 0.084; effect size = −1.490), carbon metabolism (*p* = 3.3 × 10^−2^; FDR_*p* = 0.088; effect size = −1.471), biosynthesis of secondary metabolites (*p* = 3.6 × 10^−2^; FDR_*p* = 0.090; effect size = −1.455) and microbial metabolism in diverse environments (*p* = 4.05 × 10^−2^; FDR_*p* = 0.090; effect size= −1.397) (Figure 5d).

## 4. Discussion

This study analyzed the gut microbial diversity and community composition of mandarin fish reared under different culture systems and feeding regimens. Consistent with previous studies on aquatic animals, Fusobacteriota, Bacteroidota, Proteobacteria and Firmicutes were identified as the predominant phyla across all experimental groups (Figure 2) [22,23,24]. Fusobacteriota is quite common in both freshwater and saltwater fish, with high abundance in gut microbiota such as zebrafish, catfish, Rainbow trout, etc. [25,26,27]. Bacteroidetes is characterized by metabolic flexibility [28,29]. Similarly, Firmicutes can also participate in the fermentation of carbohydrates and the production of SCFAs [30,31,32]. The abundance of the phylum Proteobacteria in the guts of carnivorous fish seems to have a role in the digestion and absorption of proteins [33,34], and its abundance and composition changes are often regarded as important biomarkers of intestinal microecological balance [35].

A comparison was made between two experimental groups in the same pond environment (CE vs. CS, with fixed aquaculture environment and different feed types). In the pond aquaculture system, replacing live bait with compound feed increases the abundance of carbohydrate treated bacterial communities (Bacteroidetes and Firmicutes), while the abundance of Proteobacteria remains stable. This distribution feature presents a correlation with the nutrient composition of artificial feed, indicating that the protein ingredients in formulated feed can match the nutritional demands of mandarin fish. At the genus level, the core bacteria of the two groups are Cetobacterium, which is a common core microbial communities in the intestinal tract of fish. This taxon is closely related to protein fermentation and amino acid utilization, and can synthesize vitamin B12 to support host growth and energy metabolism [31,36]. The CE group has seven dominant genera with relative abundance exceeding 5%. The diverse microbial community corresponding to live bait feeding may help the host adapt to the constantly changing environmental fluctuations in the pond. The CS group had six dominant genera, except for the genus Cetobacterium, the proportion of other top bacterial genera has increased compared to CE. The Muribaculaceae can inhibit the colonization of certain bacteria, enhance intestinal barrier function, and participate in the synthesis of various amino acids [37,38]. This indicates that the CS group’s artificially formulated feed has a balanced nutritional ratio. Although there is a slight reduction in the number of dominant species, it increases the overall abundance of functional symbiotic bacteria and stabilizes the intestinal microbiota structure. Alpha diversity analysis revealed that CS had higher Chao1, ACE, Shannon and Simpson indices than CE, demonstrating that pond culture combined with artificial feed sustained richer, more balanced intestinal microbiota. Beta diversity showed no significant overall community differentiation between CE and CS, indicating that feed substitution exerted weak effects on gut microbiota under identical pond culture conditions.

LEfSe analysis further identified nine genus-level differential taxa between the two pond groups. Most taxa enriched in CS have been reported to support intestinal homeostasis in published literature: Agathobacter is associated with short-chain fatty acid synthesis and host metabolic homeostasis [39,40]; Oscillibacter correlates with cholesterol metabolism and can alleviate inflammatory responses and oxidative stress [41,42,43]; UCG-010 has a positive impact on certain specific diseases and is associated with immune and antioxidant functions [44,45,46]. The enrichment of Macellibacter in the CE group has been reported to correlate with high-fat diets [47], which reflects the difference in lipid content between live bait and artificial feed. PICRUSt2 can be used to predict and analyze potential functional changes in the bacterial communities of the CE and CS groups. The original *p*-values of the four tertiary pathways were low (AMPK signaling pathway, glycosphingolipid biosynthesis lactose and neolactose series, steroid degradation, and cyano amino acid metabolism), but after FDR correction, the differences were no longer significant (FDR-*p* > 0.05). Therefore, this result is only for reference and cannot confirm the corresponding metabolic changes in gut microbiota.

Under the same artificial feed regimen, obvious differences in microbial composition were observed between pond (CS) and industrial RAS groups (GS). The relative abundance of Bacteroidota, Firmicutes and Proteobacteria declined, whereas the proportion of Fusobacteriota increased in the RAS group. Such community variation may be driven by unique abiotic conditions of closed recirculating aquaculture systems. At the genus level, Cetobacterium remains the core microbiota shared by CS and GS, but the composition of the other dominant microbiota has undergone significant changes. Compared to the six dominant genera with relatively uniform abundance distribution in the CS group, the number of dominant genera in the GS group decreased to two, meaning Cetobacterium was the most dominant bacterial genus. The abundance of Aeromonas genus has decreased significantly, and related research reports suggest that some bacteria in aquaculture may have conditional pathogenic potential, leading to diseases [48]. The gut microbiota cultured in RAS may reduce the risk of this disease, but the significant simplification of microbial community structure may also reduce interspecies competition and immune regulatory stimulation among different microorganisms. The low-diversity microbiota of GS was closely associated with intensive closed industrial culture. Consistent with the results of community composition analysis, the alpha diversity of GS is lower than that of CS, and the Simpson index shows significant differences. The β diversity analysis revealed an extremely significant community difference (*p* < 0.01) between CS and GS. The above results indicate that within the scope of comparison in this group, the transformation of open ponds to closed factory circulating water significantly reduced the species richness and community uniformity of the gut microbiota of mandarin fish.

LEfSe analysis with LDA-score evaluation was conducted to screen differential taxa between CS and GS groups (Figure 4b). A total of 43 differential taxonomic units were identified between GS and CS groups, including 22 at the genus level. Eighteen genera were enriched in CS and four genera were enriched in GS. The top 10 genera contributing most to group differences were Cetobacterium, Moraxella, Olsenella, Alloprevotella, NK4A214_group, ASF356, Lachnospiraceae_NK4A136_group, Oscillibacter, Romboutsia and Turicibacter. From the genus-level relative-abundance pattern across individual samples, Cetobacterium showed distinctly higher relative abundance in GS samples and acted as the highest-contributing biomarker for the RAS group. Previous studies have confirmed that Cetobacterium can synthesize vitamin B12 and participate in the production of short-chain fatty acids [49,50]. By contrast, Moraxella, Olsenella, Oscillibacter, and other genera maintained higher abundance across individual samples of the CS group. The results indicated that pond culture supported more diverse gut-microbial members, whereas RAS culture simplified the intestinal-microbial composition of mandarin fish. PICRUSt2 prediction analysis showed that Level-1 pathways of metabolism and genetic-information processing, together with subsequent top-ranked Level-2 and Level-3 pathways related to gene transcription, translation, amino-acid synthesis and carbon metabolism, had low raw-*p* values but yielded FDR-*p* values between 0.075 and 0.090 after correction. Although the low original *p*-value suggests a potential predictive trend of increased microbial genetic information related processes and changes in carbon utilization patterns under RAS conditions, these inferred changes cannot be considered as actual in vivo metabolic changes in the gut microbiota after FDR correction.

Explanation of the Limitations of Grouping Design: This study used three treatment groups (CE: pond live bait; CS: artificial feed for ponds; GS: RAS artificial feed). Due to the fact that RAS–live bait is not the mainstream breeding mode in the actual breeding process, there was a lack of RAS–live bait groups and a complete 2 × 2 factorial design was not formed. Therefore, this study cannot completely decouple the independent main effects and the interaction effects of cultural environment and feed type. All comparative conclusions in this study are only applicable to the two pairs of comparisons that can be achieved in the existing three groups: (1) CE vs. CS (fixed pond environment, variable feed); (2) CS vs. GS (fixed artificial feed, variable culture environment). We cannot quantify the interaction term between environment and diet, nor can we compare live bait vs. artificial feed under RAS conditions.

## 5. Conclusions

This study analyzed the differential characteristics of feeding mode and aquaculture environment on the structure, diversity, and functional pathways of gut microbiota in mandarin fish. Research has found that both the cultivation environment and the type of feed have an impact on gut microbiota. Within pond systems, replacing live bait with artificial compound feed induced mild shifts in intestinal microbial composition without altering overall community structure. When fish are fed the same compound feed, the shift from pond to industrial RAS was accompanied by simplified gut microbial communities, reduced alpha diversity, and dramatic shifts in dominant bacterial taxa, resulting in distinct community separation between the two groups. While LEfSe identified multiple differential taxa in both pairwise comparisons, PICRUSt2 functional predictions revealed no statistically robust intergroup KEGG pathway differences after FDR correction. Therefore, this part of the results is only for reference. This work illuminated the divergent gut microbial adaptive responses of mandarin fish to feed and aquaculture environment, providing microecological theoretical support for optimizing healthy breeding management of mandarin fish.

## Figures and Tables

**Table 1:** Alpha diversity of gut microbiota in three groups.

	Chao1	Ace	Shannon	Simpson
CE group	208.31 ± 26.93	208.02 ± 28.00	4.51 ± 0.35	0.86 ± 0.027
GS group	211.94 ± 22.96	211.92 ± 23.80	3.49 ± 0.45	0.71 ± 0.06
CS group	262.43 ± 17.04	264.45 ± 16.76	4.79 ± 0.42	0.87 ± 0.04

## Data Availability

The authors declare that all data supporting the conclusions of this study are available within the article.

## References

[1] Zheng J., Yao Y., Rui Q., Zhou Y., Li F., Jiang W., Chi M., Liu S., Cheng S., Chen J. (2024). Effect of different feeding regimens on physiological indicators, intestinal transcriptome, and bacterial flora of mandarin fish (*Siniperca chuatsi*). Comp. Biochem. Physiol. Part D Genom. Proteom..

[2] Liu M., Jiang Y., Sun W., Zhang H. (2025). The current situation and countermeasures of the development of the mandarin fish industry. Curr. Fish..

[3] Che S., Chen J., Zhang H., Xu W., Li Y., Dan X., Mo Z. (2025). Impacts of live and artificial feed on histology, biochemical indicators, gene expression, and bacterial resistance in mandarin fish (*Siniperca chuatsi*). Fish Shellfish Immunol..

[4] Jia S., Lu M., Lan T., Gao Z., Ding J., Meng R., Wang X., Wu X., Huang L., Li X. (2025). Effects of exercise intensity on large yellow croaker (*Larimichthys crocea*): A comprehensive analysis of growth, physiology, muscle fiber structure, and gut health. Aquaculture.

[5] Wei H., Tan B., Yang Q., Mao M., Lin Y., Chi S. (2023). Growth, nonspecific immunity, intestinal flora, hepatopancreas, and intestinal histological results for *Litopenaeus vannamei* fed with diets supplement with different animal by-products. Aquac. Rep..

[6] Ma F., Wang L., Huang J., Chen Y., Zhang L., Zhang M., Yu M., Jiang H., Qiao Z. (2023). Comparative study on nutritional quality and serum biochemical indices of common carp (*Cyprinus carpio*) aged 11 to 13 months aged cultured in traditional ponds and land-based container aquaculture systems. Food Res. Int..

[7] Nugraha M.A.R., Dewi N.R., Awaluddin M., Widodo A., Sumon M.A.A., Jamal M.T., Santanumurti M.B. (2023). Recirculating Aquaculture System (RAS) towards emerging whiteleg shrimp (*Penaeus vannamei*) aquaculture. Int. Aquac. Res..

[8] Irani A., Agh N. (2020). Rainbow trout larvae production in an airlift-based recirculating system. Aquaculture.

[9] Liu C., Wang L.R., Xu J.X., Zheng J.J., Xu Y.Y., Jin Z., Feng D., Zhang M., Yu M., Jiang H. (2025). Physiological and immune responses of largemouth bass (*Micropterus salmoides*) under the regulation of exercise intensity: An integrated perspective of blood, liver and intestinal analysis. Aquaculture.

[10] Schmidt V., Amaral-Zettler L., Davidson J., Summerfelt S., Good C. (2016). Influence of fishmeal-free diets on microbial communities in Atlantic salmon (*Salmo salar*) recirculation aquaculture systems. Appl. Environ. Microbiol..

[11] Nicholson J.K., Holmes E., Kinross J., Burcelin R., Gibson G., Jia W., Pettersson S. (2012). Host-gut microbiota metabolic interactions. Science.

[12] Flint H.J., Duncan S.H., Scott K.P., Louis P. (2015). Links between diet, gut microbiota composition and gut metabolism. Proc. Nutr. Soc..

[13] Luan Y., Li M., Zhou W., Yao Y., Yang Y., Zhang Z., Ringø E., Olsen R., Clarke J., Xie S. (2023). The Fish Microbiota: Research Progress and Potential Applications. Engineering.

[14] Nossa C.W., Oberdorf W.E., Yang L.Y., Aas J.A., Paster B.J., DeSantis T.Z., Brodie E.L., Malamud D., Poles M.A., Pei Z. (2010). Design of 16S rRNA gene primers for 454 pyrosequencing of the human foregut microbiome. World J. Gastroenterol..

[15] Callahan B.J., McMurdie P.J., Rosen M.J., Han A.W., Johnson A.J.A., Holmes S.P. (2016). DADA2: High-resolution sample inference from Illumina amplicon data. Nat. Methods.

[16] Bolyen E., Rideout J.R., Dillon M.R., Bokulich N.A., Abnet C.C., Al-Ghalith G.A., Alexander H., Alm E.J., Arumugam M., Asnicar F. (2019). Reproducible, interactive, scalable and extensible microbiome data science using QIIME 2. Nat. Biotechnol..

[17] Chao A. (1984). Non-parametric estimation of the classes in a population. Scand. J. Stat..

[18] Hill T.C.J., Walsh K.A., Harris J.A., Moffett B.F. (2003). Using ecological diversity measures with bacterial communities. FEMS Microbiol. Ecol..

[19] Simpson E.H. (1997). Measurement of Diversity. J. Cardiothorac. Vasc. Anesth..

[20] Langille M.G.I., Zaneveld J., Caporaso J.G., McDonald D., Knights D., Reyes J.A., Clemente J.C., Burkepile D.E., Thurber R.L.V., Knight R. (2013). Predictive functional profiling of microbial communities using 16S rRNA marker gene sequences. Nat. Biotechnol..

[21] Douglas G.M., Maffei V.J., Zaneveld J., Yurgel S.N., Brown J.R., Taylor C.M., Huttenhower C., Langille M.G. (2019). PICRUSt2: An improved and extensible approach for metagenome inference. bioRxiv.

[22] Ingerslev H.C., Jorgensen L.V.G., Strube M.L., Larsen N., Dalsgaard I., Boye M., Madsen L. (2014). The development of the gut microbiota in rainbow trout (*Oncorhynchus mykiss*) is affected by first feeding and diet type. Aquaculture.

[23] Liu W.S., Wang W.W., Ran C., He S.X., Yang Y.L., Zhou Z.G. (2016). Effects of dietary scFOS and lactobacilli on survival, growth, and disease resistance of hybrid tilapia. Aquaculture.

[24] Kanika N.H., Liaqat N., Chen H.F., Ke J., Lu G.Q., Wang J., Wang C.H. (2025). Fish gut microbiome and its application in aquaculture and biological conservation. Front. Microbiol..

[25] Bledsoe J.W., Peterson B.C., Swanson K.S., Small B.C. (2016). Ontogenetic characterization of the intestinal microbiota of channel catfish through 16S rRNA gene sequencing reveals insights on temporal shifts and the influence of environmental microbes. PLoS ONE.

[26] Michl S.C., Ratten J.M., Beyer M., Hasler M., LaRoche J., Schulz C. (2017). The malleable gut microbiome of juvenile rainbow trout (*Oncorhynchus mykiss*): Diet-dependent shifts of bacterial community structures. PLoS ONE.

[27] Stagaman K., Burns A.R., Guillemin K., Bohannan B.J. (2017). The role of adaptive immunity as an ecological filter on the gut microbiota in zebrafish. ISME J..

[28] Tailford L.E., Crost E.H., Kavanaugh D., Juge N. (2015). Mucin glycan foraging in the human gut microbiome. Front. Genet..

[29] Johnson E.L., Heaver S.L., Walters W.A., Ley R.E. (2017). Microbiome and metabolic disease: Revisiting the bacterial phylum Bacteroidetes. J. Mol. Med..

[30] Mountfort D.O., Campbell J., Clements K.D. (2002). Hindgut fermentation in three species of marine herbivorous fish. Appl. Environ. Microbiol..

[31] Tsuchiya C., Sakata T., Sugita H. (2010). Novel ecological niche of *Cetobacterium somerae*, an anaerobic bacterium in the intestinal tracts of freshwater fish. Lett. Appl. Microbiol..

[32] DiBaise J.K., Zhang H., Crowell M.D., Krajmalnik-Brown R., Decker G.A., Rittmann B.E. (2008). Gut microbiota and its possible relationship with obesity. Mayo Clin. Proc..

[33] Rajili S.M., Smidt H., Vos W.M.D. (2010). Diversity of the human gastrointestinal tract microbiota revisited. Environ. Microbiol..

[34] Ray A.K., Ghosh K., Ring E. (2012). Enzyme-producing bacteria isolated from fish gut: A review. Aquac. Nutr..

[35] Shin N.R., Whon T.W., Bae J.W. (2015). Proteobacteria: Microbial signature of dysbiosis in gut microbiota. Trends Biotechnol..

[36] Chen C.Z., Li P., Liu L., Li Z.H. (2022). Exploring the interactions between the gut microbiome and the shifting surrounding aquatic environment in fisheries and aquaculture: A review. Environ. Res..

[37] Chung Y.W., Gwak H.J., Moon S.M., Rho M., Ryu J.H. (2020). Functional dynamics of bacterial species in the mouse gut microbiome revealed by metagenomic and metatranscriptomic analyses. PLoS ONE.

[38] Li S.L., Qian Q.F., Yang H., Wu Z.L., Xie Y.S., Yin Y., Cui Y., Li X.L. (2024). Fucoidan alleviated dextran sulfate sodium-induced ulcerative colitis with improved intestinal barrier, reshaped gut microbiota composition, and promoted autophagy in male C57BL/6 mice. Int. J. Mol. Sci..

[39] Hua X.Y., Zhu J., Yang T., Guo M., Li Q., Chen J., Li T.Y. (2020). The gut microbiota and associated metabolites are altered in sleep disorder of children with autism spectrum disorders. Front. Psychiatry.

[40] Liu G.S., Song Y., Yan J.S., Chai Y.J., Zhao Y.F., Ma H. (2025). Identification of enterotype for patients with Alzheimer’s disease. J. Transl. Med..

[41] Li C.H., Stražar M., Mohamed A.M.T., Pacheco J.A., Walker R.L., Lebar T., Zhao S.J., Lockart J., Dame A., Thurimella K. (2024). Gut microbiome and metabolome profiling in Framingham heart study reveals cholesterol-metabolizing bacteria. Cell.

[42] Konikoff T., Gophna U. (2016). Oscillospira: A central, enigmatic component of the human gut microbiota. Trends Microbiol..

[43] Martinez J.A. (2024). Mediterranean diet and olive oil redox interactions on lactate dehydrogenase mediated by gut Oscillibacter in patients with long-COVID-19 syndrome. Antioxidants.

[44] Yang J.P., Li Y.N., Wen Z.Q., Liu W.Z., Meng L.T., Huang H. (2021). Oscillospira a candidate for the next-generation probiotics. Gut Microbes.

[45] Ali Q., Ma S., Farooq U., Liu B.S., Wang Z.C., Sun H., Cui Y.L., Li D.F., Shi Y.H. (2024). Chronological dynamics of the gut microbiome in response to the pasture grazing system in geese. Microbiol. Spectr..

[46] Mei S.H., He G.X., Chen Z., Yang C.J., Wu X.R., Zhu M.M., Xu D.H., Wang K.G., Wang C.M., Zhu E.P. (2025). Fermented distiller’s grains improve broiler immunity and antioxidant capacity by modulating cecal microbiota and cecal/plasma metabolomes. BMC Microbiol..

[47] Lim T., Lee K., Kim R.H., Cha K.H., Koo S.Y., Moon E.C., Hwang K.T. (2022). Black raspberry extract can lower serum LDL cholesterol via modulation of gut microbial composition and serum bile acid profile in rats fed trimethylamine-N-oxide with a high-fat diet. Food Sci. Biotechnol..

[48] Guo C., Zhu G.Q., Wang Y.K. (2003). Isolation, identification, and optimal treatment drug screening of 14 strains of Aeromonas bacteria from diseased aquatic animals. Fish. Sci..

[49] Navarrete P. (2012). PCR-TTGE analysis of 16S rRNA from rainbow trout (*Oncorhynchus mykiss*) gut microbiota reveals host-specific communities of active bacteria. PLoS ONE.

[50] Xie M.X., Zhou W., Xie Y.D., Li Y., Zhang Z., Yang Y.L., Olsen R.E., Ran C., Zhou Z.G. (2021). Effects of *Cetobacterium somerae* fermentation product on gut and liver health of common carp (*Cyprinus carpio*) fed diet supplemented with ultra-micro ground mixed plant proteins. Aquaculture.

